# Research progress of ferroptosis in Parkinson’s disease: a bibliometric and visual analysis

**DOI:** 10.3389/fnagi.2023.1278323

**Published:** 2023-11-14

**Authors:** Yangguang Lu, Yiqun Chen, Zihan Jiang, Yaoying Ge, Ruotong Yao, Shangze Geng, Jinxiu Zhang, Feng Chen, Yukai Wang, Guangyong Chen, Dehao Yang

**Affiliations:** ^1^The First School of Medicine, School of Information and Engineering, Wenzhou Medical University, Wenzhou, China; ^2^The Second School of Medicine, Wenzhou Medical University, Wenzhou, China; ^3^Department of Neurology, The Third Affiliated Hospital of Wenzhou Medical University, Wenzhou, China; ^4^Department of Neurology, The Second Affiliated Hospital, Zhejiang University School of Medicine, Hangzhou, China

**Keywords:** ferroptosis, iron-dependent cell death, Parkinson disease, bibliometrics, biomedical research

## Abstract

**Background:**

In recent years, the role of ferroptosis in Parkinson’s disease (PD) has become a research hotspot based on evidence of abnormal iron deposition and lipid peroxidation damage in the brains of PD patients. This study aims to examine the relevant research on ferroptosis and PD from a bibliometric perspective.

**Methods:**

Original research and review articles related to ferroptosis and PD were retrieved from the Web of Science Core Collection (WOSCC) database. Statistical analysis and visualization of information including countries, institutions, authors, journals, and keywords of the included studies were conducted using the R software package “bibliometrix.”

**Results:**

A total of 414 articles met the inclusion criteria, averaging 37.86 citations per article. From 2012 to 2022, the average annual growth rate of research in this area was 63.44%. The corresponding authors of published articles were mainly affiliated with institutions in China, the United States, and Australia. Shanghai Jiao Tong University in China and the University of Melbourne in Australia emerged as the most active and influential institutions. The journal with the highest H-index and publication output was *Free Radical Biology and Medicine*. “Ferroptosis,” “immunotherapy,” “prognosis” and “microenvironment” were identified as high-frequency keywords, indicating current and future research directions in this field.

**Conclusion:**

This bibliometric study provides insights into current research hotspots and emerging trends in the growing field of ferroptosis research related to PD. The high-frequency keywords identified highlight active areas of investigation involving methods, mechanisms, and populations of interest.

## 1. Introduction

Parkinson’s disease (PD) has been established as the second most common neurodegenerative disease, following Alzheimer’s disease (AD; Reich and Savitt, 2019). The clinical manifestations of PD encompass various motor impairments, such as postural instability, resting tremor, bradykinesia, and rigidity, as well as non-motor symptoms, including anxiety, depression, psychosis, and cognitive impairment, which can potentially advance to dementia (Fearnley and Lees, 1991). The main pathological features of PD are the loss of dopaminergic neurons in the substantia nigra pars compacta, the formation of intracellular Lewy bodies, and the progressive degeneration of dopaminergic neurons associated with systemic iron accumulation (Schneider and Obeso, 2015; Erkkinen et al., 2018; Shahmoradian et al., 2019). Currently, there is no cure for PD, and symptomatic relief can only be achieved through medication such as levodopa, dopamine agonists, and dopamine metabolism inhibitors to enhance dopamine neurotransmission (Connolly and Lang, 2014; Erkkinen et al., 2018). Considering the economic and healthcare burdens imposed by PD (Kalia and Lang, 2015), it is crucial to develop new targets to improve current diagnostic and treatment methods for PD patients.

Iron metabolism plays a critical role in the pathogenesis of neurodegenerative diseases (Ward et al., 2014). Although iron is essential for cell proliferation and survival, excessive iron accumulation increases dopamine oxidation and induces the loss of dopaminergic neurons (Healy et al., 2017). Studies have suggested that the elevated iron levels may contribute to the aggregation of α-synuclein, a hallmark of neuronal fiber damage in PD (Mehra et al., 2019). Furthermore, previous research has identified iron as a potential target for the diagnosis and treatment of PD (Moreau et al., 2018). Ferroptosis, a recently discovered form of cell death involving iron-dependent protein degradation (Gao et al., 2016), has been associated with the reduced activity of glutathione peroxidase 4 (GPX4) and the accumulation of reactive oxygen species on membranes (Wu et al., 2018). Ferroptosis is characterized by the increased iron ion load and the extensive lipid peroxidation (Hou et al., 2016), both of which are present in the brains of PD patients (Mahoney-Sánchez et al., 2021). In recent years, there has been increasing attention on the role of ferroptosis in PD research. Therefore, understanding the research progress and trends regarding the role iron plays in the pathogenesis of PD is very necessary.

Bibliometric analysis is a method used to evaluate the characteristics of published scientific research, particularly in specific scientific fields (Ellegaard and Wallin, 2015). Its aim is to identify the key features of relevant publications (Durieux and Gevenois, 2010). Compared to other traditional research methods, bibliometric analysis harnesses visualization tools to analyze published academic literature, providing more comprehensive insights and offering significant advantages (Kokol et al., 2021). Importantly, bibliometric analysis can gage the academic impact of publications, highlight emerging hotspots and cutting-edge areas, and assist researchers in discerning historical emphases and trends within specific research topics. It stands as a pivotal indicator guiding future follow-up studies in the field.

The role of ferroptosis in the pathogenesis of PD has become a research hotspot during the past 5 years. However, no bibliometric analysis has hitherto been conducted in this field. To compile compelling evidence and provide valuable insights for the early diagnosis, prevention, and treatment of PD, we conducted a bibliometric analysis.

## 2. Materials and methods

### 2.1. Data collection

Relevant studies on the genetic factors of PD pathogenesis were extracted from the Web of Science Core Collection (WOSCC) database. The selection was limited to the Science Citation Index Expanded (SCI-EXPANDED) and Social Sciences Citation Index (SSCI), which are widely used sources in bibliometric analysis. The Web of Science database was selected due to its extensive coverage of past literature, as it offers a rich pool of materials for analysis (Yeung, 2019). Furthermore, it boasts higher accuracy in journal categorization compared to other databases, further enhancing the reliability of our data and findings (Wang and Waltman, 2016). We conducted a search using the keywords “ferroptosis” and “Parkinson” with the following search strategy: TS = (Parkinson* OR PD) AND TS = (Ferroptosis OR “Iron Death” OR “iron-dependent cell death”). We retrieved all relevant studies indexed up to Oct 16, 2023, without language restrictions. Additionally, we excluded types of studies including meeting abstract, editorial materials, news item, and letters. The bibliometric data were exported as plain text files, including full records and cited references. No automation tools were used during the search. The data collection and statistical processes were conducted independently by two researchers, and any discrepancies were resolved through discussion.

### 2.2. Data analysis

We used Microsoft Excel software to record information such as titles, institutions, sources (journals or book chapters), authors, references, disciplines, and keywords. Data analysis was performed using the Bibliometrix package in R version 4.2.3 software (Ross Ihaka, Robert Gentleman) since it is a commonly used comprehensive tool for bibliometric mapping analysis (Aria and Cuccurullo, 2017). We utilized this tool to conduct the following data analysis and generate figures and tables: (1) statistics on author productivity and impact; (2) statistics on the most frequently cited articles; (3) statistics on research sources (journals or books) and determination of core journals using Bradford’s law (Weinstock, 1971); (4) statistics on high-frequency keywords, trends, and correlations using heatmaps, line graphs, ternary plots, and network graphs; (5) statistics on inter-institutional collaboration; (6) reference citation analysis. Additionally, we used the online bibliometric analysis tool (accessed on Oct 16, 2023)^1^ to analyze publication trends and international collaboration, and created the visualizations.

## 3. Results

### 3.1. General characteristics of publications

**Figure 1:**
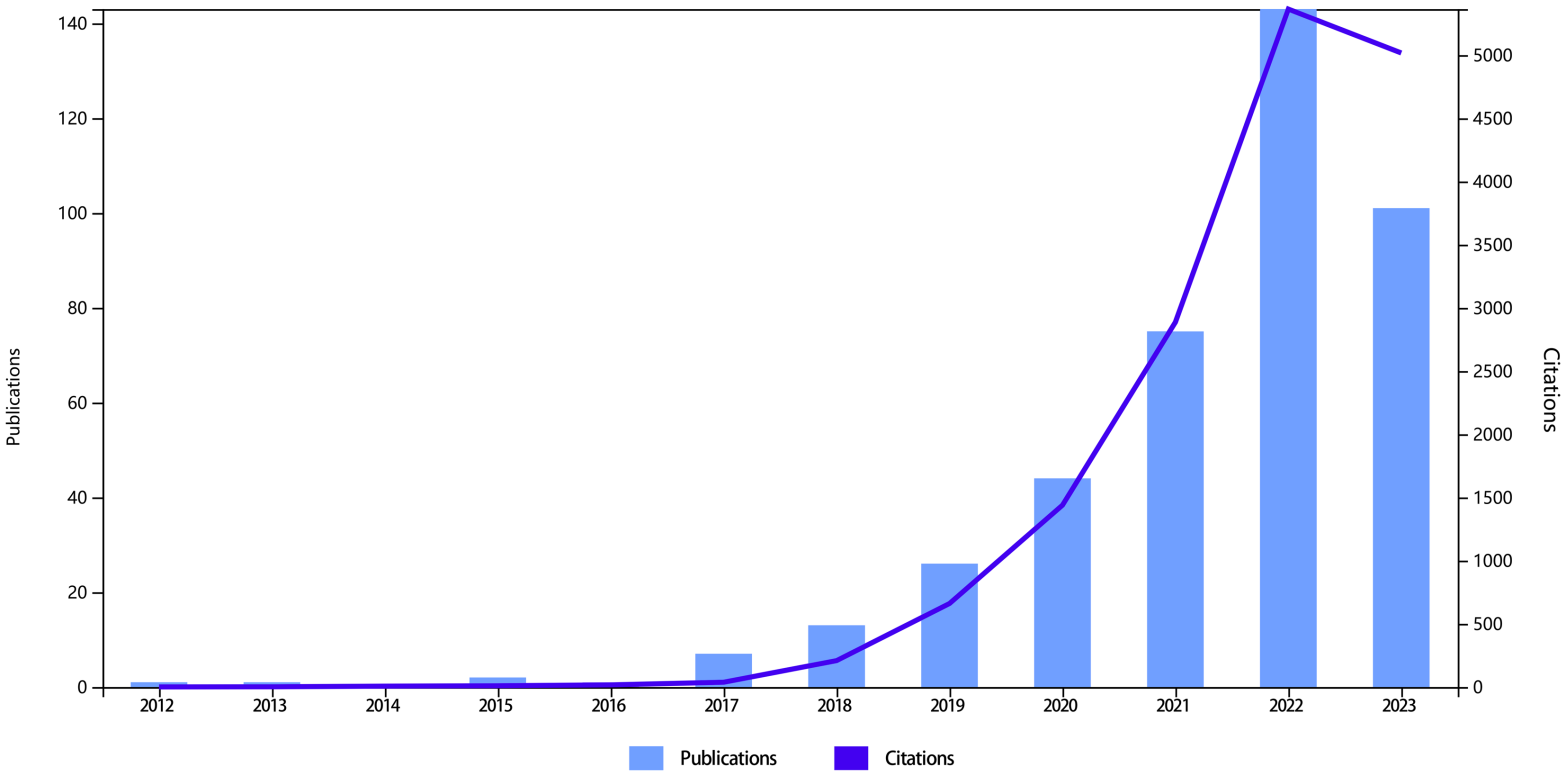
Number of publications and citations per year for studies of iron death in Parkinson’s disease.

A total of 424 research articles on the correlation between ferroptosis and PD were published from 2012 to 2023. After excluding meeting abstracts (*n* = 5), editorial materials (*n* = 4), and letter (*n* = 1), a total of 283 original articles (68.36%) and 131 reviews (31.64%) were included. Overall, these 414 studies involved 2,580 authors and 912 keywords, with 25,233 references from 215 sources (journals or books). Additionally, the average number of citations per article was 37.86, with an average of 7.89 authors per article. The average age of the documents was 1.59 years, and the proportion of international collaborative research accounted for 16.91% of studies. From 2012 to 2022, the average annual growth rate of research was 63.44%, with a significant surge in the number of publications and citation frequency over the past 5 years (Figure 1).

### 3.2. Author analysis

**Figure 2:**
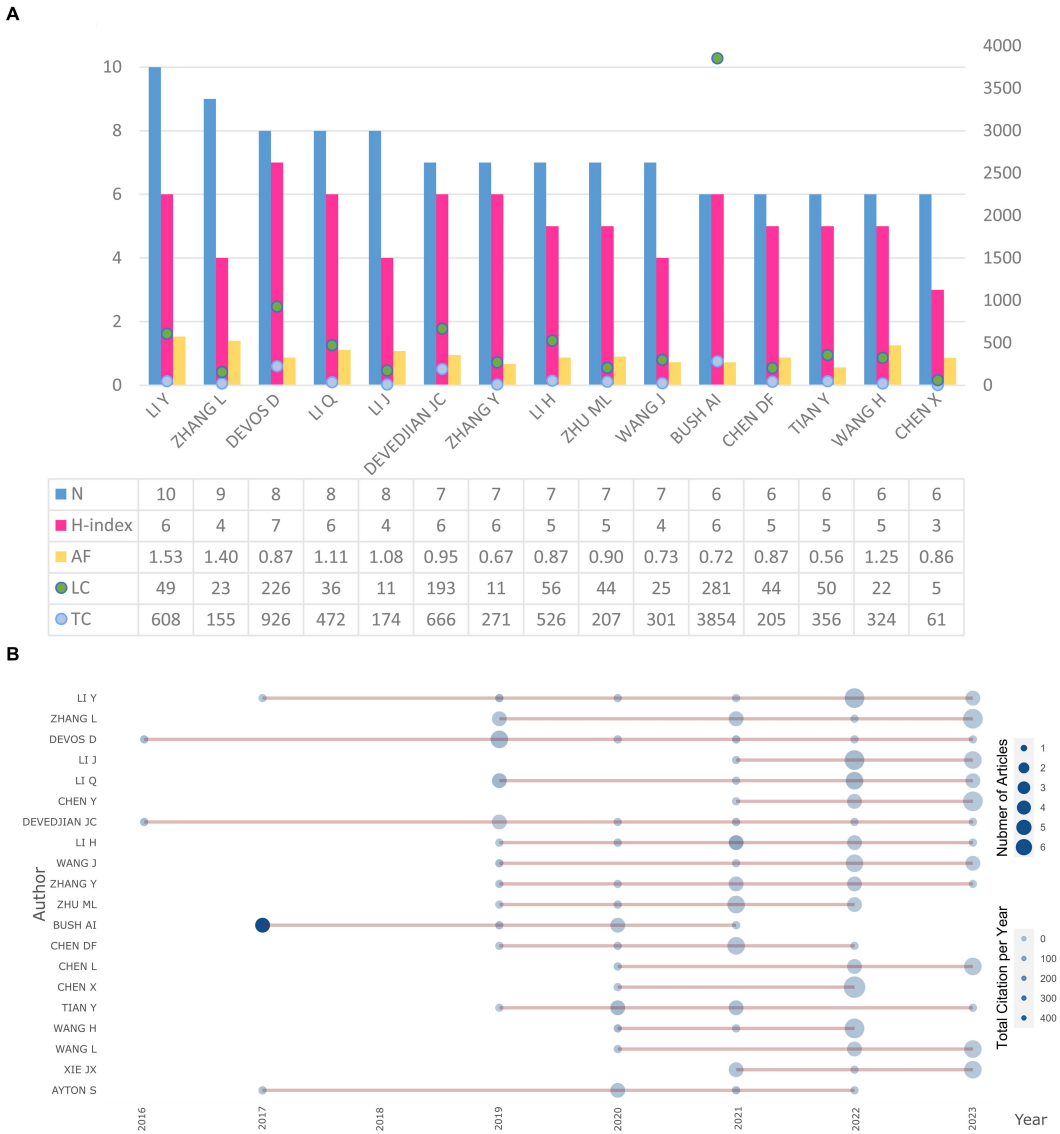
Information of the most produced authors on related research. **(A)** Plots of the 10 most produced authors’ H-index and citation data; **(B)** Productivity over time of the 20 most productive authors. Abbreviations: AF, Articles Fractionalized; TC, Total Citation; LC, Local Citation.

We summarized 15 most productive authors in the field of ferroptosis and PD correlation research (Figure 2A). These authors were from China (*n* = 12), France (*n* = 2) and Australia (*n* = 1). Li from China have the highest number of publications (*n* = 10). The H-index is a hybrid quantitative indicator to evaluate research output based on the publication quantity and the citation count (Bornmann and Daniel, 2007). Devos ranked first among all authors (H-index = 7). Additionally, Conrad from Germany held the highest total citation count (TC) among all authors, with 4,122 citations, followed closely by Bush AI from Australia (TC = 3,854). Bush led in total citation count among the top 15 authors with the highest publication productivity. Besides, Local Citation (LC) reflects the citation count of an author’s articles within a specific field, with Bush boasting the highest LC value (LC = 281). The Articles Fractionalized (AF) indicator is typically used to measure author productivity and partially quantify research achievements, with Li from China holding the highest AF value (AF = 1.53) in the present study.

We summarized the trends in publication productivity and citation counts over time for the top 20 influential authors involved in ferroptosis and PD correlation research (Figure 2B). The size of the nodes represents the publication productivity at different time points, while the depth of color reflects the citation count at those points. Notably, articles authored by Bush in 2017 has garnered the highest total citation count, offering valuable insights for subsequent research. Devos and Devedjian have both been active in this field of research from 2016 to 2023, with their publication productivity peaking in 2019. Chinese authors like Zhang exhibited an increasing trend in publication productivity after 2019. However, considering that the depth of node color remained relatively consistent, there has been no significant variation in the citation count of articles published by all authors in recent years.

### 3.3. Top cited papers

The top 10 most cited papers in the field of ferroptosis and its correlation with PD was identified (Table 1). Out of these 10 papers, four were categorized as “Article” and six as “Review.” They were published between 2017 and 2021, originating from the United States (*n* = 2), China (*n* = 2), Australia (*n* = 2), the United Kingdom (*n* = 1), France (*n* = 1), and Finland (*n* = 1). The lowest TC among them was 82 (Zhang et al., 2020). The review titled “Ferroptosis: A Regulated Cell Death Nexus Linking Metabolism, Redox Biology, and Disease” by Stockwell et al. (2017) from the United States, published in *Cell* in 2017, had the highest number of both field-specific citations (LC = 151) and total citations (TC = 3,241). In second place in terms of field-specific citations (LC = 128) and total citations (TC = 403) was an article titled “Ferroptosis, a newly characterized form of cell death in Parkinson’s disease that is regulated by PKC,” authored by Do Van et al. (2016) from France in 2016. Both highly cited articles focused on the significant role of ferroptosis in neurodegenerative diseases and the potential therapeutic benefits of manipulating ferroptosis to improve the treatment of neurodegenerative diseases, particularly PD (Do Van et al., 2016; Stockwell et al., 2017). Normalized total citations (NTC) can eliminate the differences in citation counts arising from different fields and publication years. Among the articles, the review titled “Ferroptosis and its potential role in the physiopathology of Parkinson’s Disease” by Mahoney-Sánchez et al. (2021) from France, published in *Prog Neurobiol* in 2021, has the highest NTC (11.70). This review focused on elucidating the pathogenic role of α-synuclein in Parkinson’s disease, its connections to ferroptosis-mediated cell death, and the efficacy and mechanisms of the iron chelator deferiprone in clinical investigations, providing insights with potential translational relevance.

**Table 1:** Top 10 most locally cited papers on related research.

SCR	Title	Type	Year	TC	LC	NTC	Journal	FA	Country	DOI
1	Ferroptosis: A Regulated Cell Death Nexus Linking Metabolism, Redox Biology, and Disease	Review	2017	3241	151	4.07	Cell	Stockwell BR	United States	10.1016/j.cell.2017.09.021
2	Ferroptosis, a newly characterized form of cell death in Parkinson’s disease that is regulated by PKC	Article	2016	403	128	1.00	Neurobiol Dis	Do Van B	France	10.1016/j.nbd.2016.05.011
3	Ferroptosis and cell death mechanisms in Parkinson’s disease	Review	2017	226	71	1.91	Neurochem Int	Guiney SJ	Australia	10.1016/j.neuint.2017.01.004
4	Ferroptosis and its potential role in the physiopathology of Parkinson’s Disease	Review	2021	166	43	11.70	Prog Neurobiol	Mahoney-Sanchez L	France	10.1016/j.pneurobio.2020.101890
5	Striking while the iron is hot: Iron metabolism and ferroptosis in neurodegeneration	Review	2019	260	33	4.74	Free Radic Biol Med	Masaldan S	Australia	10.1016/j.freeradbiomed.2018.09.033
6	Ferroptosis was more initial in cell death caused by iron overload and its underlying mechanism in Parkinson’s disease	Article	2020	82	31	4.32	Free Radic Biol Med	Zhang P	China	10.1016/j.freeradbiomed.2020.03.015
7	Alpha synuclein aggregation drives ferroptosis: an interplay of iron, calcium and lipid peroxidation	Article	2020	111	26	3.62	Cell Death Differ	Angelova PR	United Kingdom	10.1038/s41418-020-0542-z
8	Targeting Nrf2 to Suppress Ferroptosis and Mitochondrial Dysfunction in Neurodegeneration	Review	2018	240	25	3.01	Front Neurosci	Abdalkader M	Finland	10.3389/fnins.2018.00466
9	FTH1 Inhibits Ferroptosis Through Ferritinophagy in the 6-OHDA Model of Parkinson's Disease	Article	2020	130	25	3.48	Neurotherapeutics	Tian Y	China	10.1007/s13311-020-00929-z
10	Ferroptosis and Its Role in Diverse Brain Diseases	Review	2019	263	24	3.45	Mol Neurobiol	Weiland A	United States	10.1007/s12035-018-1403-3

Abbreviations: SCR, Standard competition ranking; TC, Total Citation; LC, Local Citation; NTC, Normalize Total Citation; FA, First Author.

### 3.4. Journal analysis

**Figure 3:**
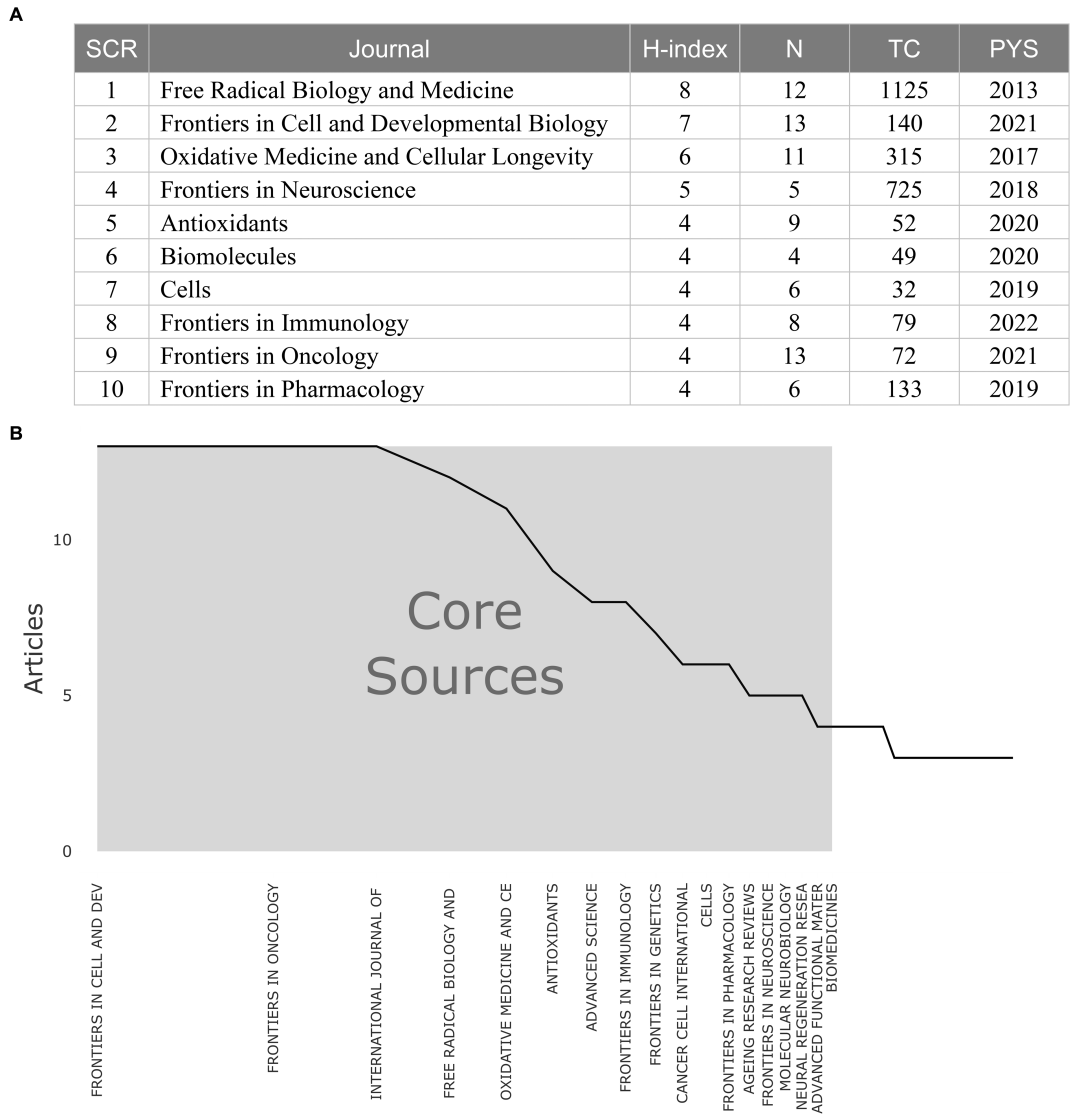
Information and data from sources (journals or book chapters) from relevant research. **(A)** Top 10 sources with the highest H-index on related research. **(B)** Core sources identified by Bradford’s law. Abbreviations: SCR, Standard competition ranking; N, Number of Articles; TC: Total Citation; PYS: Publication year start.

We summarized the total citations and publication output from the top 10 sources (including journals and book chapters) with the highest H-index in the field of ferroptosis-induced cell death and its correlation with Parkinson’s disease (Figure 3A). Among these sources, the journal *Free Radical Biology and Medicine* had the highest H-index (H-index = 8) and total citations (TC = 1,125), ranking first among all 215 sources. The journals *Frontiers in Oncology* (*n* = 13) and *Frontiers in Cell and Developmental Biology* (*n* = 13) had the highest publication output. Among the top 10 sources with the highest H-index in this field, five journals belonged to the Frontiers publishing group. According to Bradford’s law, among the 215 sources, 18 journals, such as *Frontiers in Oncology*, *Frontiers in Cell and Developmental Biology*, and *Free Radical Biology and Medicine*, are identified as the core journals due to their relatively high publication output (Figure 3B). Among them, seven journals belonged to the Frontiers publishing group.

### 3.5. Trend topics and keywords analysis

**Figure 4:**
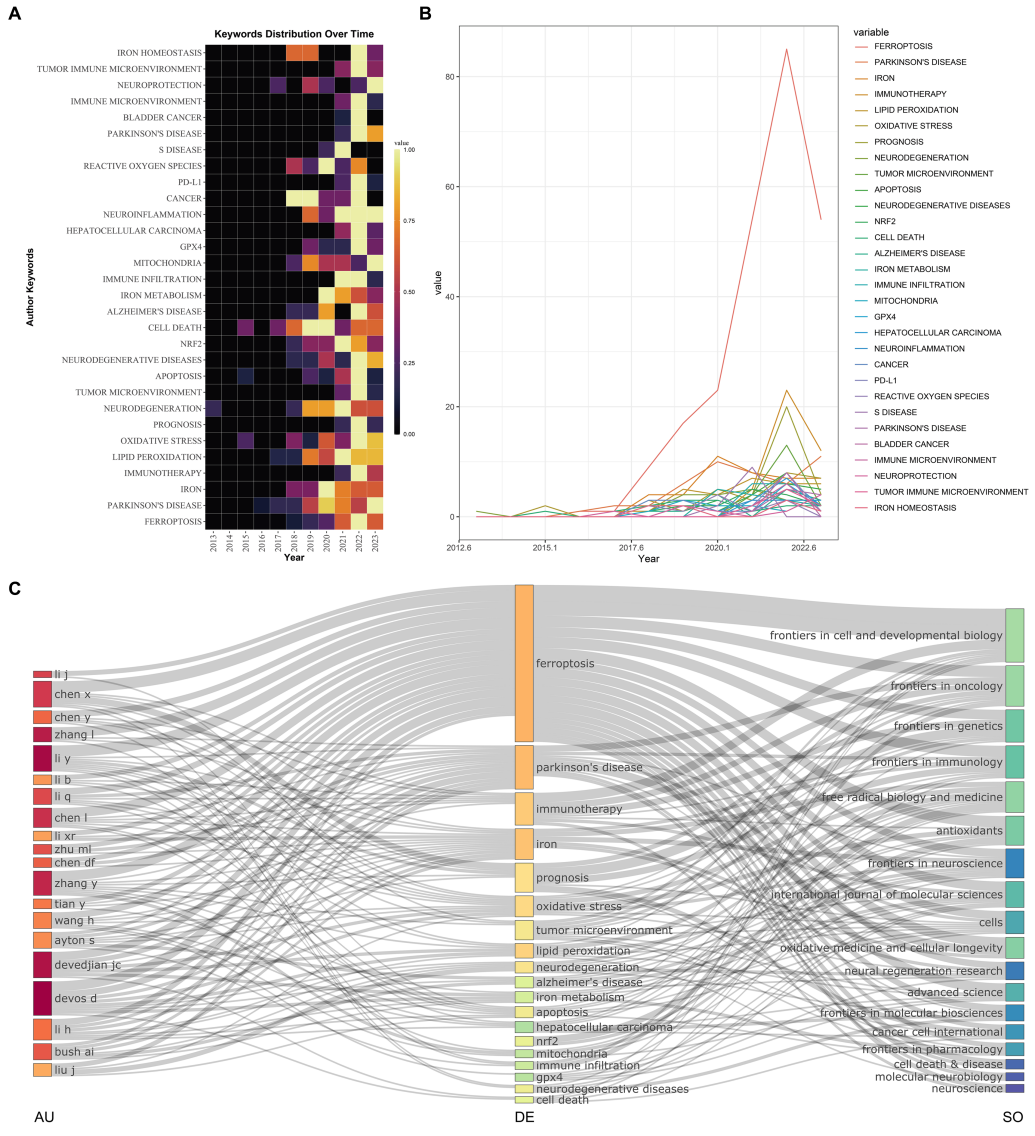
Information of keywords on relevant research. **(A)** Heatmaps of author keywords in different time periods; **(B)** Line chart of author keywords in different time periods; **(C)** Three-field plots between authors, keywords, and sources. Abbreviations: AU, Author; DE, Keywords; SO, Sources.

We summarized the usage patterns and trends of author keywords in the field of ferroptosis and its correlation with PD at different time points (Figures 4A,B). After 2018, the frequency of keyword usage increased. Among them, “Ferroptosis” emerged as the most frequently used keyword after 2017. Its usage has witnessed a substantial surge, indicating a growing research focus on this specific area. Following closely behind were the keywords “Immunotherapy,” “Prognosis” and “Tumor Microenvironment,” which have gained a high popularity in the past 5 years. Over the last 3 years, the usage of keywords such as “Tumor Immune Microenvironment,” “Oxidative Stress,” “Neuroinflammation,” “Neuroprotection” and “Mitochondria” has shown a significant upward trend, suggesting their potential as emerging research hotspots.

The co-occurrence relationships between keywords, authors, and publication sources were presented in a Three-Field Plot (Figure 4C). The size of each node represented its frequency or importance, while the connections between nodes represented co-occurrence relationships between two indicators, with the thickness of the lines indicating the frequency or strength of the co-occurrence. From left to right, the three fields each represent authors, keywords, and relevant journals. The strongest co-occurrence relationship existed between the keyword “ferroptosis” and the journal *Frontiers in Cell and Developmental Biology*. Apart from “ferroptosis” and “Parkinson’s disease,” among the most important keywords, “immunotherapy” is mainly used by the journal *Frontiers in Cell and Developmental Biology*, while “prognosis” is primarily used by the journal *Frontiers in Genetics*. Significant authors show strong co-occurrence with the keyword “ferroptosis.” Notably, certain authors exhibited strong co-occurrence with specific keywords; for example, Chen was closely linked with “prognosis,” Liu with “neurodegeneration,” and both Devedjian and Devos with “lipid peroxidation.” These observations shed light on influential authors and teams in this research domain and provide insights into potential future research directions. Journals such as *Frontiers in Genetics*, *Frontiers in Immunology*, and *Free Radical Biology and Medicine* show a correlation with the hot keywords “prognosis” and “oxidative stress.” It was worth noting that these journals were considered core publications according to Bradford’s law.

**Figure 5:**
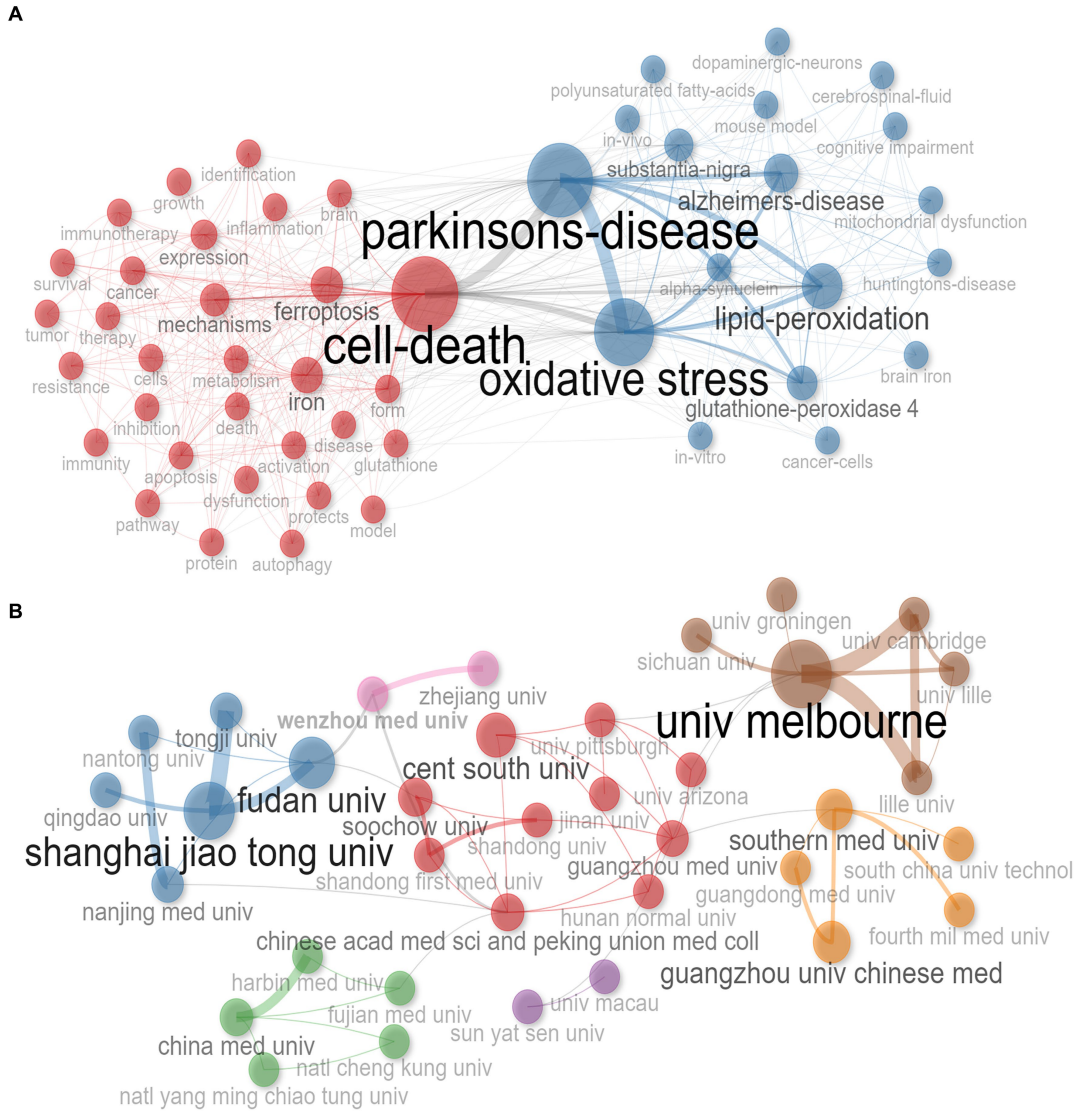
Network analysis on relevant research. **(A)** Research topics clustered by mapping co-occurrences of keywords; **(B)** Scientific collaboration between institutions.

The interrelationships between keywords were presented in a network diagram (Figure 5A). The size of each node represented the frequency of usage for that keyword, while the thickness of the connections between nodes indicates the correlation between keywords. Significantly, keywords can be divided into two main clusters. Among all the keywords, “Parkinson’s disease” shows the strongest correlation with “Oxidative stress.” Furthermore, “Parkinson’s disease” was also strongly correlated with “Alzheimer’s disease,” “Lipid peroxidation” and “Glutathione peroxidase 4.” This finding underscored the current research focused on investigating the relationship between PD and other neurodegenerative disorders. “Parkinson’s disease” and “Cell death” act as a bridge between the two clusters, exhibiting the highest centrality with the largest node area. Additionally, “Oxidative stress” and “Lipid peroxidation” also exhibited a strong centrality.

### 3.6. Institution analysis

To assess the importance and collaboration among institutions, we generated a network plot (Figure 5B). The size of each node represents the significance and activity level of the respective institution, while the thickness of the connecting lines indicates the level of collaboration between institutions. The institutions involved in ferroptosis-PD research were categorized into seven major clusters, with the majority located in China. The most important and active institutions were Melbourne University, followed by Shanghai Jiao Tong University, Fudan University, Guangzhou Medical University, and Central South University. Among the top five institutions, apart from Melbourne University in Australia, the other four were all located in China. In terms of collaboration, Fudan University and Tongji University demonstrated the highest degree of co-occurrence. While Melbourne University exhibited significant co-occurrence with Cambridge University and Lille University. The institution with the highest centrality was Guangzhou Medical University, followed by Beijing Union Medical College Hospital of the Chinese Academy of Medical Sciences and Central South University. These institutions served as the core organizations facilitating collaboration among different groups. Additionally, Zhejiang University and Wenzhou Medical University exhibited notable differences in their distribution and structure within the co-occurrence network. They had the highest clustering coefficient among all institutions (Cluster = 8), suggesting that they possess unique research characteristics, advantages, or distinct research directions and issues. Despite these collaborative networks, regional constraints in institutional collaborations were evident, with Shanghai Jiao Tong University primarily collaborating with Tongji University and Fudan University, while Wenzhou Medical University primarily collaborated with Zhejiang University.

### 3.7. Country or territory analysis

We summarized the top 10 countries in terms of citation count for research articles on the correlation between ferroptosis and PD, based on the country of affiliation of the corresponding authors (Table 2). China ranked first in both citation count (TC = 5,846) and the number of publications (*n* = 281), followed by the United States closely follows with a citation count of 5,036 from 35 publications, and Australia with a citation count of 1,263 from 14 publications. Although China had a significantly higher number of publications than other countries, it ranked last among the top 10 countries in terms of average article citations (AAC). In contrast, the United States ranked first with an average of 143.90 citations per article, followed by Australia (AAC = 90.20) and France (AAC = 81.50). The inconsistencies in publication count, total citation count, and average citation count per article may be attributed to heterogeneity in the quality of research articles among these countries/regions.

**Table 2:** Top 10 most cited countries on related research.

SCR	Country	TC	Freq	N	SCP	MCP	AAC
1	China	5,846	933	281	257	24	20.80
2	United States	5,036	179	35	25	10	143.90
3	Australia	1,263	33	14	5	9	90.20
4	Germany	816	52	11	6	5	74.20
5	Japan	528	18	8	7	1	66.00
6	France	489	37	6	2	4	81.50
7	Canada	403	11	5	2	3	80.60
8	United Kingdom	325	30	4	2	2	81.20
9	Belgium	201	14	4	3	1	50.20
10	Spain	142	9	4	2	2	35.50

Abbreviations: SCR, Standard competition ranking; TC, Total Citation; Freq, Number of all articles considering the collaboration with other countries; N, Number of articles with a corresponding author from a certain country; SCP, Single Country Publications; MCP, Multiple Country Publications; AAC, Average Article Citations.

**Figure 6:**
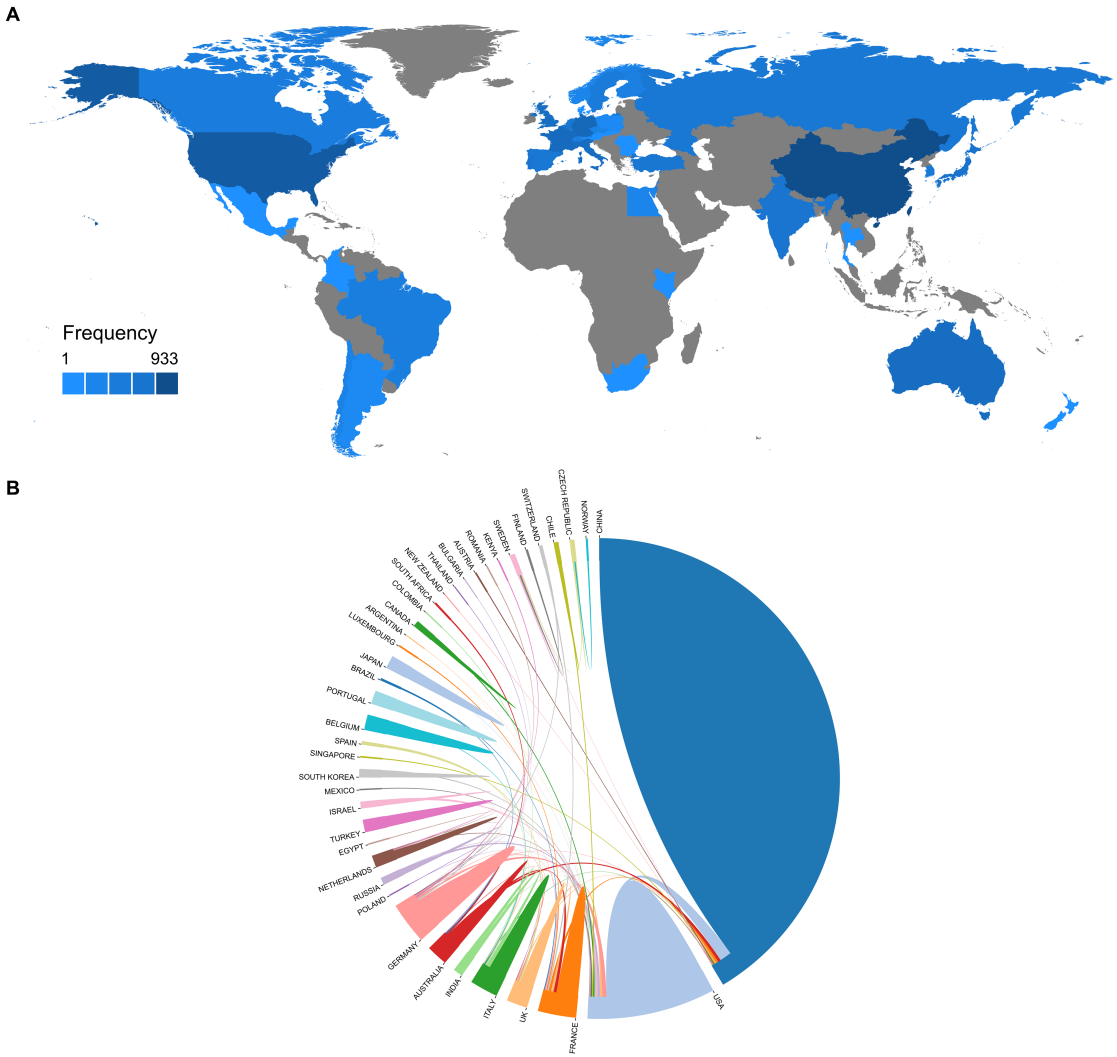
Information of countries on relevant research. **(A)** Distribution of countries contributing to the field. **(B)** Countries’ collaboration world map.

We next analyzed the publication frequencies of different countries, including co-authored papers (Figure 6A). China and the United States had the highest publication frequencies. However, research on the correlation between ferroptosis and PD was mainly conducted in North America, South America, Europe, East Asia, and Australia, with limited data from Central Asia and Africa. The collaboration relationships among different countries are presented in a network diagram (Figure 6B). In this field, China and the United States showcased extensive collaboration, resulting in 14 joint studies. China also engaged in close collaborations with institutions in Australia (*n* = 5) and Germany (*n* = 4). Additionally, Australia maintained significant collaborations with the United Kingdom (*n* = 5), while France also collaborated with the United Kingdom (*n* = 5).

### 3.8. Reference analysis

We summarized the top 10 most cited references among the included studies on the correlation between ferroptosis and PD (Table 3). Among the top 10 articles, seven were articles, and three were reviews, with citation counts ranging from 71 to 263. The corresponding authors were from the United States (*n* = 5), Germany (*n* = 2), China (*n* = 1), France (*n* = 1), and Australia (*n* = 1). The most cited reference is an article titled “Ferroptosis: an iron-dependent form of nonapoptotic cell death” by Dixon et al. (2012), published in 2012, with a total citation count of 263. This article provided the first systematic description of ferroptosis as a novel form of nonapoptotic cell death, highlighting its hallmark feature of iron-dependent lipid peroxidation accumulation and its significant implications for neurodegenerative diseases, especially for PD. Furthermore, the study titled “CD8+ T cells regulate tumor ferroptosis during cancer immunotherapy” by Wang et al. (2019) from the United States, published in 2019, was the most recent among the top 10 articles and holds significant contemporary relevance. This study provided the latest evidence regarding “ferroptosis-specific lipid peroxidation.” Notably, among the top 10 cited references, the three most cited articles were published in *Cell*, highlighting their crucial reference value for subsequent research endeavors.

**Table 3:** Top 10 most locally cited references on related research.

SCR	Title	Type	FA	Year	TC	Journal	Country	DOI
1	Ferroptosis: an iron-dependent form of nonapoptotic cell death	Article	Dixon SJ	2012	263	Cell	United States	10.1016/J.CELL.2012.03.042
2	Ferroptosis: A Regulated Cell Death Nexus Linking Metabolism, Redox Biology, and Disease	Review	Stockwell BR	2017	151	Cell	United States	10.1016/J.CELL.2017.09.021
3	Ferroptosis, a newly characterized form of cell death in Parkinson's disease that is regulated by PKC	Article	Do Van B	2016	128	Neurobiol Dis	France	10.1016/J.NBD.2016.05.011
4	Regulation of ferroptotic cancer cell death by GPX4	Article	Yang WS	2014	127	Cell	United States	10.1016/J.CELL.2013.12.010
5	CD8+ T cells regulate tumor ferroptosis during cancer immunotherapy	Article	Wang WM	2019	100	Nature	United States	10.1038/S41586-019-1170-Y
6	Inactivation of the ferroptosis regulator Gpx4 triggers acute renal failure in mice	Article	Angeli JPF	2014	94	Nat Cell Biol	Germany	10.1038/NCB3064
7	ACSL4 dictates ferroptosis sensitivity by shaping cellular lipid composition	Article	Doll S	2017	82	Nat Chem Biol	Germany	10.1038/NCHEMBIO.2239
8	Ferroptosis: process and function	Review	Xie Y	2016	76	Cell Death Differ	China	10.1038/CDD.2015.158
9	Oxidized arachidonic and adrenic PEs navigate cells to ferroptosis	Article	Kagan VE	2017	75	Nat Chem Biol	United States	10.1038/NCHEMBIO.2238
10	Ferroptosis and cell death mechanisms in Parkinson’s disease	Review	Guiney SJ	2017	71	Neurochem Int	Australia	10.1016/J.NEUINT.2017.01.004

Abbreviations: SCR, Standard competition ranking; FA, First Author; TC, Total Citation.

## 4. Discussion

This study presents the first bibliometric analysis of the correlation between ferroptosis and PD. Out of the 414 publications retrieved from the WOSCC database, approximately 70% were original research articles, while the remaining 30% were review articles. These results indicate a significant increase in the number of relevant publications, especially after 2019, during the period from 2012 to 2023. It is highly conceivable that the abundance of related publications will continue to increase in the coming years, highlighting the sustained research interest in the correlation between ferroptosis and PD.

The total citation count serves as an important indicator of interest within a specific research field (Seglen, 1997). In this study, the top 10 most cited articles all investigated the correlation between ferroptosis and PD. These articles examined the mechanisms, diagnostic markers, and therapeutic targets of ferroptosis in PD from diverse perspectives. They revealed the significant roles of iron metabolism abnormalities, phospholipid oxidation accumulation, mitochondrial dysfunction, and α-synuclein deposition in PD. Iron-induced cell death of dopaminergic neurons in the substantia nigra-striatum pathway leads to the dopamine deficiency and motor disorders. Ferroptosis has been associated with the abnormal aggregation of α-synuclein, disturbances in iron metabolism, inhibition of glutamate transporter, and depletion of glutathione and GPX4, among other factors (Guiney et al., 2017). These newly proposed pathogenic mechanisms can provide novel references and inspirations for future research on the prevention and treatment of PD. For example, iron chelators can protect neurons in PD patients from oxidative stress and mitochondrial damage by inhibiting iron-induced cell death. They can also influence the expression and clearance of α-synuclein, reducing its aggregation and toxicity in neurons (Zeng et al., 2021). These studies suggest new diagnostic biomarkers and potential treatments for PD, such as iron chelators, and ferroptosis inhibitors or inducers, providing new insights into the molecular mechanisms and therapeutic targets of PD.

The correlation between ferroptosis and PD represents a novel area of research. In addition to the most widely cited basic research studies, recent investigations provide new perspectives on the pathogenesis and clinical translation of PD. In a previous study, ferroptosis emerged as a significant contributor to neuroinflammation in PD patients, offering valuable insights for translational strategies in PD treatment (Ko et al., 2021). Moreover, researchers identified significant correlations between ferroptosis-related genes such as *DDIT4*, *RGS4*, *RELA*, and *CAV1* with PD through the detection of differentially expressed genes (DEGs; Jian et al., 2022). They employed a bioinformatics approach to elucidate the genetic correlation between PD and ferroptosis. On the clinical front, the latest research indicates that biomarkers associated with ferroptosis, such as LPINI and TNFAIP3, can be used for early diagnosis and prognosis assessment of PD (Xing et al., 2023). Furthermore, certain investigations into therapeutic drugs have suggested that iron death inhibitors like DFP and Cu(II)ATSM (Wang et al., 2022), as well as botanical extracts such as Puerarin (Wu et al., 2021), possess the capability to protect dopaminergic neurons, thereby slowing or reversing the progression of PD. These recent research directions and advancements hold significant promise for clinical translation.

To characterize publication avenues for research on ferroptosis in Parkinson’s disease, journal contributions were analyzed from multiple dimensions, and Bradford’s law was used to identify core journals in this field. The TC metric indicates a journal’s influence, while the H-index provides a quantitative measure of both publication volume and citation count (Bornmann and Daniel, 2007). *Free Radical Biology and Medicine* held the top position in both the H-index and TC dimensions. This journal primarily publishes cutting-edge research on the role of oxidative reactions in diseases. And it has made significant contributions to the field of ferroptosis and PD by publishing high-quality papers on molecular mechanisms, diagnostic methods, and treatment strategies related to ferroptosis. *Frontiers in Cell and Development Biology* is the journal with the highest publication volume and, along with six other Frontiers series journals, was identified as a core journal by Bradford’s law. Frontiers Publishing is an open-access publisher dedicated to promoting scientific progress and has several leading research journals in the fields of neuroscience, biomedical science, molecular biology, and more. These journals have made important contributions to the field of ferroptosis and PD. The core journals identified by Bradford’s law also hold considerable potential for future research in this field, providing valuable references for future researchers. However, it is important to consider that this law only takes the volume of publications in relevant categories into account without considering the research quality and impact (Weinstock, 1971). Therefore, it is necessary to integrate research findings published in various journals to gain a better understanding of the correlation between ferroptosis and PD.

Interestingly, although China published the most papers in this field, followed by the United States, the articles published by the United States had the highest ACC, indicating a high level of research quality. Additionally, the University of Melbourne in Australia demonstrated the highest importance and activity in this field, and many research institutions in China have actively participated in collaborative efforts. Generally, a country’s economic condition can influence the level and focus of research funding support, thus affecting its academic capabilities. Therefore, it is imperative for research institutions across regions to engage in extensive collaboration to facilitate global research in this field and further understand the similarities and differences of ferroptosis, as well as its correlation with PD among different races.

Keywords can be considered as the core content of a specific article, and their frequency provides important clues to the main trends in a specific research field. Our research demonstrated that “Ferroptosis” has been one of the most frequently used keywords in our specific field since 2017. As a regulated cell death mechanism, ferroptosis has attracted significant interest due to its associations with degenerative diseases, carcinogenesis, stroke, traumatic brain injury, and other related phenomena (Stockwell et al., 2017). Notably, recent studies have explored clinical issues using genes associated with ferroptosis, thereby elevating the significance of “Ferroptosis” in the realm of functional genomics (Wu et al., 2023). In the field of functional genomics, there has been a growing interest in circulating tumor DNA (ctDNA) in recent years (Lin et al., 2021). ctDNA is intricately connected to cancer (Cheng et al., 2016), and the term “Tumor Immune Microenvironment” has gained prominence as a frequently used keyword. Given that our search parameters were confined to pd, it is highly conceivable that the potential correlation between “Ferroptosis” in functional genomics and “ctDNA” might have been overlooked. Presently, “ctDNA” is a widely used keyword in the field of functional genomics, suggesting that there is a burgeoning need to further investigate the role of “ferroptosis” in the broader context of genomics and its potential relevance to “ctDNA” in the field of clinical neurology.

In the present study besides “Parkinson’s disease” the most significant keyword was “oxidative stress.” oxidative stress refers to the disruption of the balance between oxidative agents and antioxidants within cells leading to excessive production of reactive oxygen species and causing damage to cells. In PD oxidative stress is considered one of the most important factors contributing to neurodegenerative damage (Sun et al., 2021). Therefore when studying the correlation between PD ferroptosis and oxidative stress in the future the role of oxidative stress should also be emphasized. Furthermore “Alzheimer’s disease” was identified as an important keyword. AD and PD are both neurodegenerative diseases and although their relationship remains largely unclear some studies suggest that ferroptosis is a common pathogenic mechanism for both diseases (Jakaria et al., 2021; Xing et al., 2023). Future researchers can use this as a starting point for conducting new studies to further elucidate the role of ferroptosis in neurodegenerative diseases.

Bibliometrics brings unique advantages for evaluating scientific research in specific fields (Ellegaard and Wallin, 2015), and our bibliometric analysis boasts the following strengths. Firstly, our study is the first bibliometric analysis in this field, providing insightful implications for future research. Secondly, our bibliometric analysis was based on the WOSCC database, which has a strict selection criteria and only included important academic journals from various disciplines. This meticulous approach guarantees the reliability and credibility of the data utilized in our study. Thirdly, WOSCC provides comprehensive citation retrieval, allowing access to the complete citation network of 1.5 billion references. This functionality enables the tracking of research impact and development trends (Wang and Waltman, 2016; Yeung, 2019). Last but not least, we conducted a comprehensive bibliometric analysis focusing on countries, institutions, journals, authors, references, and keywords. We also utilized the Bibliometrix package and online tools to visualize the data, effectively presenting the development of research in this field.

However, it is important to acknowledge some limitations of our study. Firstly, there may be high-quality publications that are not considered due to their absence from the WOSCC database. Secondly, since our retrieval was conducted in October 2023, the data coverage for the year 2023 might not be complete, which could lead to potential misunderstandings regarding research hotspots and publication trends. Despite the incomplete inclusion of 2023-related research, a considerable number of articles have been published. This indicates that the correlation between ferroptosis and PD has become a hotspot. Additionally, certain studies might not accurately identify their hotspots due to being published late. Finally, the Bibliometrix package in R language tends to favor quantitative analysis. Indeed, future research should incorporate qualitative research methods, such as interviews, to compensate for the shortcomings of quantitative research.

Conducting research on the correlation between ferroptosis and PD holds significant clinical significance. From a preventive perspective, such research helps us better understand the pathogenesis of PD and provides a foundation for identifying high-risk populations at an earlier stage. Additionally, further research in this area will contributes to the early clinical diagnosis of PD and the identification of different PD subtypes. Regarding PD treatment, the application of ferroptosis-specific inhibitors has gained momentum, given that these inhibitors can rescue neuronal death and represent new therapeutic targets for PD (Wang et al., 2022).

Our bibliometric analysis provided directions and insights for future researchers’ studies. Researchers can further explore the role and regulation of ferroptosis in the pathogenesis of PD, elucidate the interrelationships and influences between ferroptosis and other forms of cell death, as well as discover more ferroptosis-related genes and signaling pathways. Furthermore, researchers can integrate multidisciplinary data from fields such as medicinal chemistry, molecular biology, and cell biology to screen and validate the functional effects of ferroptosis inhibitors or inducers related to PD. This process can elucidate their mechanisms and advantages in PD treatment. Moreover, additional research methods can be introduced in future studies, such as systematic reviews and meta-analyses, to summarize previous research and address controversial issues (Lee, 2018). Besides, animal models and clinical trials are warranted to evaluate the impact of PD-related ferroptosis regulators on PD occurrence, development, and treatment, providing stronger evidence for precision medicine in PD. At last, there is a need for enhanced interdisciplinary, inter-institutional, and cross-regional collaborations, data and resource sharing, global research on PD pathogenesis and ferroptosis correlation. It is crucial to foster communication and innovation in PD-related research to bring better preventive and treatment strategies for PD patients.

## 5. Conclusion

The number of research papers investigating the relationship between ferroptosis and PD has been steadily increasing, resulting in the emergence of several prominent themes in this field. Collaborative efforts among institutions and across countries have played a vital role in advancing research within this domain. Institutions and researchers from various nations need to collaborate to identify relevant areas of focus and enhance our understanding of this growing and innovative subject matter.

## Data availability statement

The raw data supporting the conclusions of this article will be made available by the authors, without undue reservation.

## Author contributions

YL: Data curation, Investigation, Methodology, Visualization, Writing – original draft. YC: Data curation, Formal analysis, Methodology, Validation, Visualization, Writing – original draft. ZJ: Data curation, Formal analysis, Methodology, Validation, Visualization, Writing – original draft. YG: Data curation, Formal analysis, Validation, Writing – original draft. RY: Data curation, Formal analysis, Writing – review & editing. SG: Data curation, Writing – review & editing. JZ: Data curation, Writing – review & editing. FC: Data curation, Writing – review & editing. YW: Data curation, Writing – review & editing. GC: Conceptualization, Investigation, Project administration, Resources, Supervision, Writing – review & editing. DY: Conceptualization, Funding acquisition, Investigation, Project administration, Resources, Supervision, Writing – review & editing.

## Footnotes

^1^ https://bibliometric.com/
